# Exposure to airborne mycotoxins: the riskiest working environments and tasks

**DOI:** 10.1093/annweh/wxad070

**Published:** 2023-11-28

**Authors:** Anna Maria Marcelloni, Daniela Pigini, Alessandra Chiominto, Angela Gioffrè, Emilia Paba

**Affiliations:** Department of Occupational and Environmental Medicine, Epidemiology and Hygiene, Italian Workers’ Compensation Authority (INAIL), Via Fontana Candida 1, Monte Porzio Catone, Rome 00078, Italy; Department of Occupational and Environmental Medicine, Epidemiology and Hygiene, Italian Workers’ Compensation Authority (INAIL), Via Fontana Candida 1, Monte Porzio Catone, Rome 00078, Italy; Department of Occupational and Environmental Medicine, Epidemiology and Hygiene, Italian Workers’ Compensation Authority (INAIL), Via Fontana Candida 1, Monte Porzio Catone, Rome 00078, Italy; Department of Occupational and Environmental Medicine, Epidemiology and Hygiene, Italian Workers’ Compensation Authority (INAIL), Contrada Ficarella, Lamezia Terme, Catanzaro 88046, Italy; Department of Occupational and Environmental Medicine, Epidemiology and Hygiene, Italian Workers’ Compensation Authority (INAIL), Via Fontana Candida 1, Monte Porzio Catone, Rome 00078, Italy

**Keywords:** inhalation, molds, mycotoxin, occupational exposure, risk assessment, toxicity, working task

## Abstract

**Objectives:**

There is growing interest in the role of airborne mycotoxins in occupational environments, however, their impact on human health still remains poorly investigated. This review aims to provide a comprehensive analysis of the existing literature on the occurrence of inhalable mycotoxins in working environments to investigate which sectors and tasks are at greater risk of exposure.

**Methods:**

We have performed a systematic search in the PubMed, Scopus and Web of Science databases from 2010 to date, without limitation of geographic location.

**Results:**

Database searches yielded 350 articles. After the removal of duplicates and applying our inclusion and exclusion criteria, 31 papers remained. Results show that the most exposed workers are those engaged in activities related to animal care and management and, in particular, in feeding tasks, while harvester cleaning seems to be the activity with the highest levels of exposure in agriculture. In healthcare settings mycotoxin concentrations are low but HVAC systems can be a source of contamination and this reinforces the relevance of further studies in this sector. The most common scenario is the exposure to multiple mycotoxins with variable concentrations depending on the working environment, the products handled or the tasks performed by workers. Some authors emphasize the importance of multi-approach sampling and analysis protocols to achieve an accurate and more realistic risk characterization.

**Conclusions:**

Results brought forward by this review can be utilized by health and safety professionals to recognize activities in which workers may be potentially exposed to airborne mycotoxins and thus undertake suitable preventive and protective measures.

**What Is Important About This Paper?**

This systematic review is important because it discusses research focused on exposure to airborne mycotoxins, an area where further research is needed to elucidate the real impact on occupational health . Exposures to mycotoxins have been found to vary across industries and working tasks, but comparisons among studies are still difficult beca?use of the variety of measurement methods and mycotoxins target. These characteristics reflect the complexity of mycotoxin exposures.

## Introduction

### Structure and function of mycotoxins

Mycotoxins are toxic compounds naturally produced by filamentous fungi, better known as molds, through their secondary metabolism. Chemically, the term refers to a heterogeneous group of small and stable molecules, with a low molecular weight, mainly represented by alkaloids, cyclopeptides, coumarins, aromatic and phenolic structures, and terpenoids. To date, about 150,000 fungal species have been formally described and more than 500 mycotoxins have been characterized. However, about 30 are of relevant concern for human and animal health including aflatoxins (AFTs), ochratoxins (OTs), tricothecenes (TCTs), fumonisins (FUMs), deoxynivalenol (DON), zeralenone (ZEN), citrin (CT) and ergot alkaloids (Haque et al. 2020; Awuchi et al. 2022).

The production of mycotoxins by molds depends on intrinsic factors (species, strain, etc.) but also on agricultural practices (e.g. fungicide usage, plowing, plant resistance) and chemical-physical parameters such as temperature, humidity, pH, gaseous composition and nature of the growth substrate (Daou et al. 2021). This last parameter represents the element that, probably more than others, influences the release of mycotoxins in the environment: vegetable substrates promote the release of mycotoxins more than animal ones. In fact, high levels of contamination can be found in cereals (corn, wheat, barley, rye, etc.), oleaginous seeds (peanuts, sunflower, etc.), fresh and dried fruit (grapes, almonds, walnuts, hazelnuts, dried figs, etc.), cocoa beans and coffee as well as in some spices such as chili, pepper and ginger (World Health Organization 2023). Consequently, the various processing products of raw materials (e.g. flours for human and animal use) are also susceptible to contamination. It is estimated that approximately 25–50% of cereal products produced worldwide are significantly being contaminated with mycotoxins to a varying degree and 5–10% of which are irreversible, causing huge economic losses (Wang et al. 2018).

Mycotoxins can be present in the environment even in the absence of any visible fungi since they can resist adverse environmental factors such as extreme temperatures and can persist long after the death of the fungal species responsible for their production. These compounds are also difficult to eliminate or inactivate from the source even after being exposed to boiling or roasting processes; this means that the removal of fungi does not guarantee the absence of mycotoxins because of their chemically resistant nature. For this reason, the detection of toxigenic fungal species may not generally predict mycotoxin presence (Straumfors 2008; Daou et al. 2021).

### Routes of exposure

In the general population mycotoxins exposure occurs primarily through the food chain, via the ingestion of contaminated food (agricultural products or meat of animals fed on contaminated crops) and the resulting health effects are widely acknowledged and subjected to extensive regulatory monitoring in most western countries. In addition to the alimentary way, some people can be exposed to mycotoxins via inhalation and this occurs mainly in occupational settings or water-damaged buildings due to the growth of mycotoxigenic mold species on materials and products.

Although most mycotoxins are not volatile they can be present in airborne dust and fungal spores which act as carriers to the respiratory system. It is not elucidated if spores are the major way of mycotoxin’s release into the air. Some authors have shown that these compounds also occur on smaller particles; fungal fragments and other small particles, such as nonorganic debris, could be potential carriers of the most aerosolized mycotoxins and therefore be a cause for concern and further study (Brasel et al. 2005). This is also supported by the findings of Gareis and Gottschalk (2014) who have shown that exudates segregated by some molds contain high concentrations of mycotoxins.

Exposure to contaminated aerosol by inhalation may occur in working environments where large quantities of susceptible materials are handled and processed. Hence, the workers at risk of exposure are those engaged in food and feed industries, silos, warehouses, drivers of vehicles deputed to transport grain (e.g. truck drivers) but also operators involved in waste treatment and disposal activities as well as maintenance of machinery for agriculture, forestry and animal husbandry. Animal feed processing plants are particularly risky for mycotoxin exposure since the authorized level of concentration in this type of food is 10 times higher than it is for human food. As an example, the EU maximum levels authorized for DON in unprocessed maize is 1750 µg/kg, while it is 750 µg/kg in cereals intended for direct human consumption (Commission Regulation 2006).

### Health effects

Inhalation effects of fungal metabolites have been studied experimentally in vitro with cells of the respiratory system and ex vivo cultured tissues, elucidating the mechanisms of action for the most common toxins, and in vivo in animal models. However, there are still few epidemiological studies which are often limited to risk assessment based on single compounds or surrogates for mycotoxin exposure. The nasal passage is the primary target for several inhaled toxicants; its epithelial lining is often the first tissue to be directly injured, for example, by the spores or metabolites of Stachybotrys chartarum (Huttunen and Korkalainen 2017).

Symptoms and effects attributed to the inhalation of mycotoxins are mucous membrane irritation, damage to the epithelium, endocrine effects, systemic reactions (fever, nausea, fatigue), immunosuppression, immunotoxic and nephrotoxic effects, acute or chronic liver damage, acute or chronic central nervous system damage, reproductive outcomes and cancer. About the last one, the International Agency for Research on Cancer classified some mycotoxins as Group 1, carcinogenic to humans (Aflatoxins), and Group 2B, possibly carcinogenic to humans (Fumonisins B1, Ochratoxin A) (List of classification-IARC).

Some studies showed that mycotoxins may play a role in the development of a complex of symptoms known as Sick Building Syndrome (fever, headache and asthenia) assuming a potential risk of exposure also for indoor occupants (e.g. office workers). In addition, it has been reported that exposure to airborne mycotoxins might represent a risk for the development of allergic diseases (Kraft et al. 2021).

Although to a lesser extent, dermal contact may represent another route of exposure to mycotoxins. This is particularly relevant in working environments where the use of short-sleeved clothes is possible or when hands are in contact with solutions containing mycotoxins. In these cases, contaminated dust particles can deposit on the skin and persist in epidermal cells causing cell death and skin cancer (Boonen et al. 2012).

The severity of diseases depends on various factors including mycotoxin toxicity, duration and intensity of exposure, individual’s age and health, and the synergistic effect of other chemicals, including other metabolites (Al-Jaal et al. 2019).

### Objectives of the study

Exposure to mycotoxins occurs mainly through food, which is undoubtedly the main source; however, the relevance of the inhalation route under specific occupational conditions needs to be further investigated and more data are still needed to identify the working activities at risk.

Thus, the main goal of the present review is to identify, summarize, and discuss research focused on this issue in order to investigate whether exposure to airborne mycotoxins can be a problem for workers’ health and, in particular, which working environments and tasks are at greater risk of exposure. This paper also aims to take stock of current methodologies for assessing exposure to airborne mycotoxins and any health effects observed in the selected studies.

Our results can be used by health and safety professionals to recognize activities in which workers may be potentially exposed to high levels of airborne mycotoxins and thus undertake preventive measures including the use of appropriate personal protective equipment.

## Methodology

### Bibliographic search strategy

Firstly, the main questions were formulated in the following framework: is there scientific evidence of exposure to airborne mycotoxins in occupational environments? What are the working environments and tasks with the highest exposure levels? Based on these questions a comprehensive search strategy has been framed to find all the articles focused on the topic of study from 2010 to date.

Literature research was conducted on the main bibliographic databases (Pubmed, Scopus and Web of Science). The first author (AMM) screened the titles and abstracts of the studies retrieved during the searches for relevance. Then, two authors (AMM and DP) assessed independently the full texts of articles identified as being potentially eligible for inclusion against the predefined criteria. Any discrepancies were resolved by consensus. Supplementary Figure S1 (see Supplementary Material) shows complete search strings for each database.

We selected original and peer-reviewed manuscripts from studies conducted in working environments and identifying the tasks performed during exposure assessment. In addition to the matrix “air,” we included those manuscripts that also considered settled dust, which is part of airborne particulate matter that fell onto surfaces/equipment and that can act as a carrier of metabolites. The articles were chosen without limitation of geographic location.

Reviews, proceedings, letters to editors, and book chapters have been retrieved and carefully read but not included in the review. In addition, studies of exposure in residential environments (e.g. dwellings) and articles written in languages other than English have been excluded. Full-texts have been carefully read and all relevant information has been entered on an electronic spreadsheet consisting of the following columns: bibliographic reference (first author and year of publication), working environment, state, matrices (air, settled dust and any other matrix contextually considered by the individual authors), sampling and analytical methods, mycotoxins target, main results and relevant authors’ conclusions including observed health outcomes if reported. When information was not available, the cell was left empty.

## Results and discussion

### Data collection

**Figure 1.**
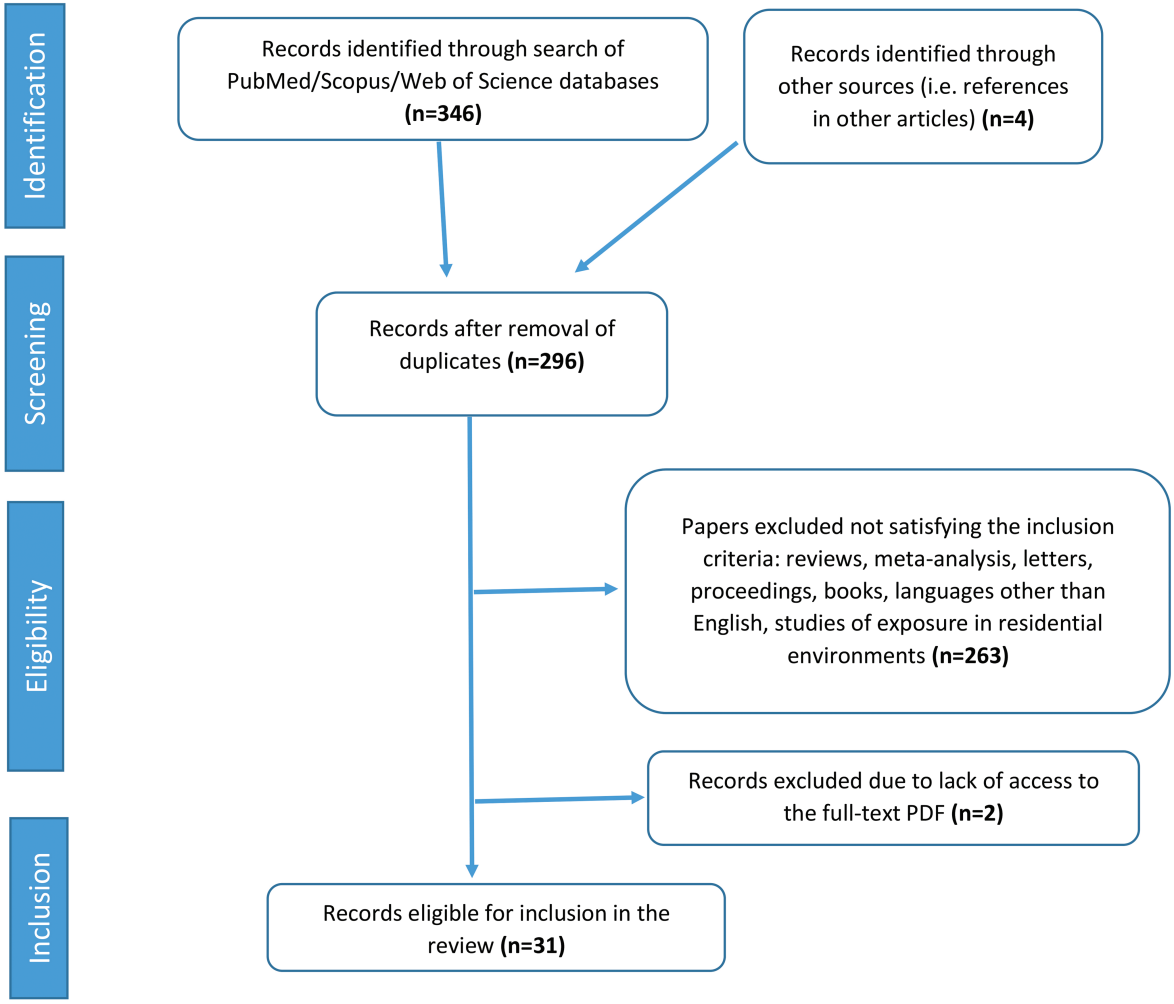
Flowchart selection process.

The bibliographic search returned a total of 346 manuscripts. Duplicates have been removed and applying the above eligibility criteria, 27 papers have remained. The references of these articles have been checked for possible important missed studies and, as a result, 4 more articles were manually included. After this, 31 manuscripts remained for systematic review. Figure 1 shows schematically the selection process of the papers.

### Characteristics of collected papers

The included studies are summarized in Table 1 where they are listed in chronological order and divided according to working areas: animal husbandry, agriculture, healthcare environments, waste/sewage treatment plants and other sectors.

**Table 1.** Extracted data from included papers (n=31)

Animal husbandry								
First author, year of publication	Working environment	State	Matrices	Sampling methods	Analytical methods	Mycotoxins	Main Results	Authors’ conclusions
Lanier et al. 2010	Cattle farms	France	Bioaerosol (ambient and personal air)	High volume sampler, 500 L/min, quartz filters; personal air pumps, 1 L/min, PC filters.	HPLC-MS	Multiple	No mycotoxins in ambient air. Gliotoxin in 3 personal filters (mean value: 2.6 mg/m^3^)	Risk of exposure by inhalation during feeding. Not all mycotoxins are aerosolized during feed handling.
Viegas et al. 2012	Poultry production	Portugal	Bioaerosol (ambient air for molds detection) Blood	Impactor, 140 L/min, Malt Extract Agar	Macroscopic, microscopic and molecular (real-time PCR) techniques (molds). ELISA (serum)	AFB1	Bioaerosol: Aspergillus flavus the most represented specie AFB_1_ in serum: average value: 2 ± 0.98 ng/ml.	Risk of exposure to AFB1 by inhalation for the presence of Aspergillus flavus and detectable levels of AFB_1_ in worker’s serum respect to the controls.
Lanier et al. 2012	Dairy cattle shed (feeding corridor)	France	Bioaerosol (ambient air)	High volume sampler, 500 L/min, quartz filters	HPLC-MS	Multiple	AFB1 and AFB2 under the quantification limits (AFB1: 0.09 ng/filter; AFB2: 0.07 ng/filter).	Mutagenity of bioaerosol: needs to continue the investigation of bioaerosol composition and mycotoxins.
Viegas et al. 2013	Swine production	Portugal	Bioaerosol (ambient air for molds detection) Settled dust Blood	Impactor, 140 L/min, Malt Extract Agar, Sterile swabbs	Microscopic observation (molds), ELISA assay (mycotoxin)	AFB1	Bioaerosol: high concentrations of A. versicolor (40.5%), Mean value of AFB1 in serum: 1.91 ± 1.68 ng/ml. Significantly higher concentrations in workers respect to the controls	Workers are at increased risk for mycotoxicosis.
Skóra et al. 2016	Poultry farms	Poland	Settled dust	Passive method	LC-MS/MS	Multiple	Aurofusarin: max value 281.44 mg/kg; Infectopyron: max value 249.12 mg/kg; Zearalenone-sulfate: max value 204.48 mg/kg; Neochinulin A: max value 784,48 mg/kg.	Weak cytotoxicity of settled dust towards chicken hepatocyte cells (range: 9.2–29.7%).
Viegas et al. 2019c	Swine production	Portugal	Bioaerosol (ambient air), waste, feed, litter Urine	Impinger Coriolis, 300 L/min	HPLC-MS	Multiple	STC in 3 air samples (<LOQ-1.42 ng/g).	The environment is adding and contributing to the workers’ total exposure to mycotoxins. This was confirmed by the biomonitoring data and the high contamination found in feed and litter.
Franco and Oliveira 2022	Feed factories Animal-producing farms (poultry-pig-dairy farms)	Brazil	Bioaerosol (ambient air) Urine	Portable aspirator, 1200 L/min, filter paper	HPLC-MS	Multiple	FUMs: range 7.85–6,839 ng/m^3^.	Biomonitoring revealed a potential health concern for OTA and FBs, although no potential health concern was observed with Probable Daily Intake (PDI) calculated through food data.
Agriculture								
Traverso et al. 2010	Laboratory for food (shelled peanuts from Vietnam)	Italy	Bioaerosol (ambient air)	Impinger, 1 L/min	HPLC	Aflatoxins	AFB1: 0.11 pg/m^3^ Low concentration of AFB2	Effectiveness of wet grinding in reducing mycotoxin concentration.
Mo et al. 2014	Sugar factory	China	Bioaerosol (ambient air) Blood	Air sampler, 20 L/min, filter paper	ELISA assay	AFB1	AFB1 not detected in air samples. Serum AFB1 albumin adducts: 51 ± 4.62 pg/mg	Only exposure to a certain level of AFB1 (>0.05 mg/Kg/die) would result in detectable levels of serum AFB1 albumin addusts.
Lai et al. 2014	Sugar and papermaking factory	China	Dust Blood		ELISA assay	AFB1	AFB1 detection: Sugarcane bagasse warehouse: 7.2 ± 1.30 µg Kg Presser 8 ± 1.23 µg Kg Paper production workshops: 8.6 ± 1.82 µg Kg AFB1 albumin adducts: range 8–212 pg/ml.	AFB1 airway exposure may be associated with a risk of hepatocellular carcinoma (HCC).
Straumfors et al. 2015b	Grain elevators	Norway	Settled dust		LC-MS/MS	Multiple	Aurofusarium: median 5,515 µg/Kg Avenaceyn Y: median 3,149 µg/Kg Culmorin: median 1,072 μg/Kg	The highest inhalational exposure came from *Fusarium* metabolites. In a worst case scenario the workers may inhale up to 10 μg/m^3^ during work shift, depending on the work intensity.
Despot Jakšić et al. 2016	Grain mill, apartments, basements	Croatia	Bioaerosol (ambient air for molds detection); dust	Mas 100 Eco air sampler, DG18.	Molecular methods, HPLC/UV-VIS	STC-producing Aspergilli	A. griseoaurantiacus (208.29 μg/mL) and A. jensenii (1.192–133.63 μg/mL) produced the highest levels of STC.	STC and the majority of STC-producing Aspergilli were cytotoxic to human lung A549 cells in relatively low concentrations suggesting that humans can be at high risk during chronic exposure.
Niculita-Hirzel et al. 2016	Grain industry	Switzerland	Bioaerosol (personal air)	Personal air pumps, 2 L/min, PTFE, clear styrene cassettes	HPLC MS/MS	Multiple	DON: 65 ng/m^3^ NIV: 59 ng/m^3^ ZEN: 3 ng/m^3^.	Cleaning procedure is a critical task.
Ferri et al. 2017	Grain mill	Italy	Dust samples Blood, urine	Static pumps (20 L/min, GF filters); high volume sampler pumps (1400 L/min, GF filter). Personal air pumps (IOM)	HPLC-FLD	AFs (M1, G2, G1, B1, B2) and aflatoxicol A (AFOH)	AFs: range: 7.2–125.4 µg/kg. Urine: AFM1 (mean: 0.035 and 0.027 ng/mL in exposed and non-exposed workers respectively, p = 0.432).	Higher AF concentration in exposed workers treating highly contaminated maize than in non-exposed controls. These differences are to be considered consistent with random fluctuations.
Jakšić et al. 2018	Grain mill	Croatia	Bioaerosol (ambient air for molds detection)	Mas 100 Eco air sampler, DG18	HPLC-MS, PCR (fum1 and fum8 genes)	FB-producing Aspergilli	Mean estimated concentration range: 22.5–1550 CFU/m^3^	No cytotoxicity of FB2 in human lung adenocarcinoma A549 and THP-1 macrophage-like cells
Viegas et al. 2019b	Bakeries	Portugal	Bioaerosol (ambient air) Settled dust	Impactor, 140 L/min, MEA and DG18 Impinger, 600 L/min Sterile swabbs	Real time PCR (moulds) HPLC-MS	Multiple	No mycotoxin in air samples; Settled dust: 6–8 mycotoxins (range: <LOQ-211 ng/g), high concentration of DON (15.95–211 ng/g)	Raw material (e.g. flour) an indoor contamination source.
Viegas et al. 2020b	Bakeries, pizzeria restaurant	Portugal	Bioaerosol (personal air)	Personal air pumps, 2 L/min (IOM, GF filter)	HPLC-MS	Multiple	DON and ZEA the most represented (range: <18–170.1 ng/g and <1.2–3.3 ng/g respectively), followed by DON-3-G, 15-AcDON, FB1, FB2, HT2, OTA, MPA and IDN.	Potential high exposure to organic dust and their constituents associated with the use of flour dust. Useful of multi-approach sampling and analytical methods.
Ndaw et al. 2021a	Grain elevators (small pilot study)	France	Bioaerosol (personal air) Urine	CIP 10-l, polyurethane foam	UHPLC–HR-MS/MS	Multiple	DON: 28.3–108 ng m^3^ AFB1: 80.0–120 pg m^3^ FB1: 97.0–873 pg m^3^ OTA: 38.0–194 ng m^3^ ZEN: 32.1–285 ng m^3^	The nature of mycotoxins and the magnitude of exposure depend on the workplace, the products handled or the tasks. Grain dust could be a source of mycotoxin exposure.
Ndaw et al. 2021b	Grain elevators (wheat and maize harvesting period)	France	Bioaerosol (personal air) Urine	CIP 10-l, polyurethane foam	UHPLC–HR-MS/MS	Multiple	DON: LOQ–80.1 ng m^3^ ZEN: LOQ–778 ng m^3^ FB1: LOQ–248 ng m^3^ T-2: LOQ–417 ng m^3^ HT-2: LOQ–2232 ng m^3^	Grain dust could be a source of mycotoxin exposure. Usefulness of multi-mycotoxin methods.
Healthcare environments								
Heutte et al. 2017	Cancer treatment center	France	Bioaerosol (ambient air)	Impinger Coriolis, 300 L/min	LC-MS/MS	Multiple	STC in 3 air samples (0.31, 0.32, 1.45 µg m^3^).	Low exposure to airborne mycotoxins
Viegas et al. 2019a	Primary Healthcare Center (PHCC)	Portugal	Bioaerosol (ambient air), HVAC filters	Impinger Coriolis, 300 L/min Sterile swabbs	HPLC-MS	Multiple	OTA: the most prevalent, <0.6–2.25 ng/ml FB2: the mycotoxin with the highest concentrations, 6 samples, <2.8–8.8 ng/ml. HVAC filters: FB2 the mostprevalent with the highest values (0.6–21.4 ng/g)	Detection of mycotoxins reinforces the relevance of studying mycotoxins in the clinical environment. HVAC system can be a source of release of mycotoxins.
Viegas et al. 2020a	Hospital	Portugal	Bioaerosol (ambient air), HVA filters	Impinger Coriolis, 300 L/min (mycotoxins)	HPLC-MS	Multiple	No mycotoxin	The study supports the importance of considering exposure to complex mixtures in indoor environments.
Waste and sewage treatment plants								
Viegas et al. 2017	Waste sorting facility	Portugal	HVAC filters (forklift cabinets)		HPLC-MS	Multiple	No mycotoxins. Most filter extracts were highly cytotoxic or medium cytotoxic.	Observing air conditioner filter replacement frequency may be a critical aspect to avoid worker’s exposure.
Schlosser et al. 2020	Waste recycling/recovery facilities	France	Bioaerosol (personal air)	CIP 10-l, polyurethane foam	UPLC-Q-Orbitrap HRMS	Multiple	AFB1: range: 0.06–0.98 ng/m^3^ STC: range: 0.01–0.98 ng/m^3^	The low levels of exposure do not suggest a significant threat to health.
Karamkhani et al. 2020	Waste processing plant (bread and plastic)	Iran	Bioaerosol (ambient and personal air) settled dust, blood	Air pumps, 2 L/min, IOM, GF filter (breathing zone and stationary position)	HPLC	AFB1	Inhalation of 4.5-15.1 ng of AFB1 in a working week. AFB1-Alb significantly higher in the exposed workers respect to controls.	The workers in handling of municipal waste may be exposed to hazardous levels of AFB1. Workers in the bread waste sorting are at greater risk.
Szulc et al. 2021	Sewage treatment plant	Poland	Bioaerosol (ambient air) Settled dust	AirPort MD8, gelatine filers (7000 L)	LC-MS/MS	Multiple	3-Nitropropionic acid and Flavoglaucin detected in the air samples (1.98 and 2.54 ng/m^3^ respectively)	Settled dust from the workstation in the sludge thickening building revealed high cytotoxicity to A-549 cells, suggesting the presence of non-biological inhalation hazards compounds which may adversely affect employees’ health.
Salambanga et al. 2022	Waste and recyclable material (urban collectors, drivers)	Canada	Bioaerosol (ambient air of the cabin)	SASS 3100 sampler, polypropylene electret microfibres, 300 L/min.	HPLC-MS	Multiple	No mycotoxins	SASS filter do not have small enough pore sizes to retain the particles that carry mycotoxins.
Szulc et al. 2022	Waste sorting plants	Poland	Bioaerosol (ambient air), settled dust	AirPort MD8, gelatine filter, (2000 L)	LC-MS/MS	Multiple	17 metabolites in air samples and 91 in settled dust, characteristics for Aspergillus, Penicillium, Alternaria, Fusarium	The most significant potential toxicity attributed to 3-nitropropionic acid whose presence was associated with neurologic illnesses.
Viegas et al. 2022b	Waste collection station (WCS) and trucks (WCT)	Portugal	Settled dust	Electrostatic dust collectors (EDC)	HPLC-MS	Multiple	No mycotoxins in EDC from WCS; settled dust filters from WCT: FB1 < LOQ (12 ng/g); settled dust samples from WCT: mycophenolic acid: range >LOQ-170.1 ng/g; STC: 6 ng/g; DON: 35 ng/g and ZEN: 6.1 ng/g	A complex exposure, particularly to fungi and their metabolites. The detection of Aspergillus section Fumigati highlights the significance of targeting this section as an indicator of occupational health risk.
Other occupational environments								
Castillo et al. 2016	Archives, libraries	Colombia	Strain collection Bioaerosol (ambient air)	Cotton swab/scalpel Mas 100 Eco air sampler	Culture and molecular methods (molds) UHPCL-QTOF LC-MS/MS (mycotoxins)	Multiple	Up to 44 mycotoxins identified. Aflatoxins, fuminisins, ochratoxin, trichothecenes, zearalenones the most frequent mycotoxins. Fungal strains did not produced these compounds.	Caution measurements are suggested to be reinforced in these settings for appropriate workers protection until more data are available.
Viegas et al. 2022a	Firefighter Headquartes’	Portugal	Bioaersol (ambient air) Passive sampling strategy: EDC, floor surface, settled dust, cloths and mops used in cleaning procedures, badges	Andersen six-stage air sampler (fungi and bacteria) Impinger Coriolis air sampler, 300 L/min Sterile swabs	HPLC-MS	Multiple	Filters: FB2, range <9 (LOQ)-9.7 ng/g. EDCs: FB2 (<9–10.4 ng/g), nivalenol < LOQ (14 ng/g), mycophenolic acid <LOQ (10 ng/g). Coriolis samples: FB2 range <6 (LOQ)-6.2 ng/g. Settled dust, cleaning cloths and mops: mycophenolic acid (< LOQ, 20 ng) and STC (<LOQ, 6 ng/g)	The multi-approach on sampling methods and laboratory assays improved data findings, enabling a more detailed and accurate risk characterization.

More than half of the collected articles (18/31) were focused on agriculture (37%) and sectors related to animal care and management (23%) confirming that these working environments are the most investigated. A percentage of 23% (7/31) was focused on waste and sewage treatment plants while 3 papers (10%) were carried out in healthcare settings and 2 in other minor sectors (libraries/archives and firefighter headquarters’).

**Figure 2.**
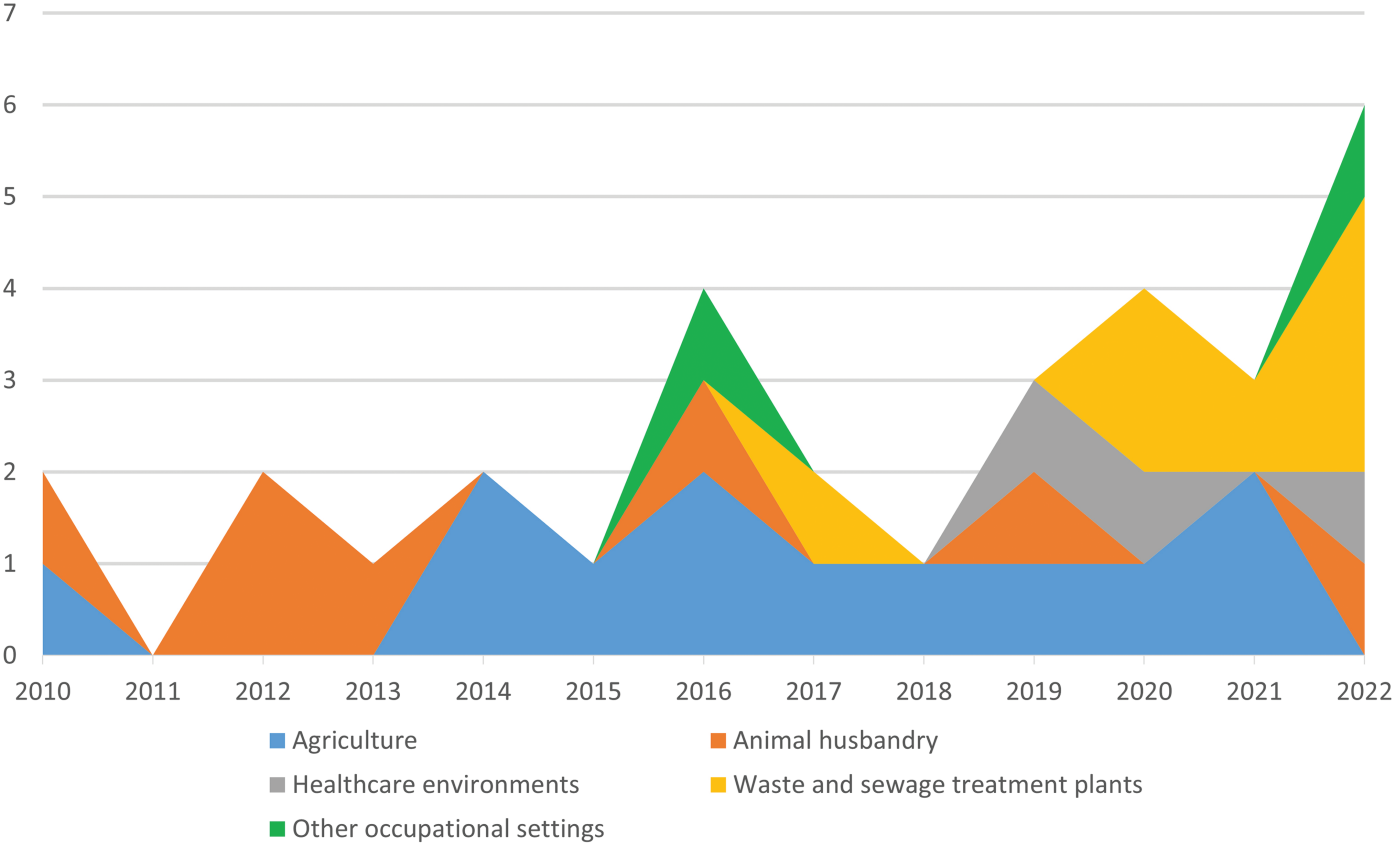
Annual distribution of collected papers according to working areas.

Despite the small size of the sample, it can be observed that until 2015 studies were focused on agriculture and animal husbandry, while from 2016 to today there is a greater diversification of the monitored working environments (Figure 2). This highlights a growing recognition and interest in the role of airborne mycotoxins as health hazards in all work environments, including those considered less typical (e.g. healthcare settings).

### Mycotoxins target

Over the years, the target of papers has changed from studying one or few metabolites at a time (AFB1 was the mycotoxin most studied) to searching for several mycotoxins across the same sample. This is certainly due to the development of analytical resources allowing the characterization of occupational exposure to different metabolites simultaneously. This approach have made it possible to observe the co-occurrence of two or more mycotoxins in most air samples while the interest in a single mycotoxin is increasingly a rare occurrence.

Table 2 reports a list of mycotoxins detected in different working environments. A great number of mycotoxins was detected in agriculture with a prevalence of Fusarium metabolites (DON, and ZEN). These compounds are well-known contaminants of wheat grain and their frequency has increased in the last 20 years probably due to more frequent rainfall episodes during wheat flowering (Krysinska-Traczyk et al. 2007). In particular, DON has been reported as the more prevalent compound in raw materials since it is common to find it, for instance, in maize and soybean meal with several consequences for animal health (e.g. increased susceptibility to infectious diseases, reactivation of chronic infection, decreased vaccine efficacy) and a huge economic impact on production (Guerre 2016).

**Table 2.** Mycotoxins detected in the different working areas.

Mycotoxins	Animal husbandry	Agriculture	Healthcare settings	Waste plants	Other environments	Number of papers
Gliotoxin	1					1
Aflatoxin B1 (AFB1)	3	4		2		9
Aflatoxin B2 (AFB2)	1					1
Aurofusarin	1					1
Infectopyron	1					1
Zearalenone-sulfate	1					1
Neochinulin A	1					1
Sterigmatocystin (STC)	1	2	1	2	1	7
Fumonisins	1					1
Aurofusarium		1				1
Avenaceyn Y		1				1
Culmorin		1				1
Deoxynivalenol (DON)		5		1		6
Nivalenol (NIV)		1			1	2
Zearalenone (ZEN)		4		1	1	6
Aflatoxin M1 (AFM1)		1				1
Aflatoxins (AFs)		1			1	2
Aflatoxicol A (AFOH)		1				1
DON-3-G		1				1
15-AcDON		1				1
Fumonisin B1 (FB1)		3		1		4
Fumonisin B2 (FB2)		1	1		1	3
HT2		2				2
Ochratoxin A (OTA)		2	1		1	4
MPA		1		1	1	3
IDN		1				1
T-2		1				1
3-Nitropropionic acid				2		2
Flavoglaucin				1		1
Other trichothecenes					1	1

Note: Number of papers (last column) indicates in how many studies the specific mycotoxin was detected.

In animal husbandry, Aspergillus-derived mycotoxins, as AFB1, seem to be predominant. Aspergillus species are widespread in nature but they occur with high frequency in food, feed and dairy products, mainly in milk and cheese (Ráduly et al. 2020).

Sterigmatocystin (STC) has been detected in all occupational settings, including the healthcare facilities. It is a carcinogenic compound released mainly by Aspergillus section Versicolores and classified by IARC as possibly carcinogenic to humans (Group 2B). Several incidences of STC contamination have been worldwide reported in food and feed but also in indoor environments such as damp mouldy dwellings and carpet dust (Despot et al. 2016).

### Sampling and analytical methods

Several sampling and analytical techniques are taken into account to assess airborne mycotoxin exposure in working environments.

The collection of settled dust represents the sampling approach most used followed by ambient air collection, by the use of impingers at different flow rates (300, 600 and 1L/min) (Traverso et al. 2010;Heutte et al. 2017; Viegas et al. 2019 a, b, c; Viegas et al. 2020a; Viegas et al. 2022a), and active impactors on nutrient media (MEA and DG18) (Viegas et al. 2012, 2013, 2019b; Despot Jakšić et al. 2016; Jakšić et al. 2018) or gelatine filters (Szulc et al. 2021, 2022).

Personal air pumps equipped with inhalable fraction selectors (IOM or CIP 10) are frequently used as they allow to evaluate tasks-based exposure during a full working day (Ferri et al. 2017; Karamkhani et al. 2020; Schlosser et al. 2020; Viegas et al. 2020b; Ndaw et al. 2021a; b).

The use of an high-efficiency dry filter air sampler (SASS 3100) to assess the exposure of urban waste collectors and drivers proved to be unsuitable as this device does not have small enough pore sizes to retain the particles that carry mycotoxins (Salambanga et al. 2022).

It is increasingly common to use passive and active-collection methods simultaneously as they provide different information: active air samples reflect the load of a shorter period corresponding to the sampling time (mostly minutes) while passive methods allow the collection of contamination over a longer period (days, weeks or months). For this reason, many authors suggest using both approaches to ensure a more accurate and realistic risk characterization (Viegas et al. 2020b, 2022; Ndaw et al. 2021b).

Among the different analytical techniques that of High Performance Liquid Chromatography (HPLC) coupled with various detectors such as UV-VIS, MS and MS/MS is the most common. The use of HPLC coupled to tandem mass spectrometry (Liquid chromatography-mass Spectrometry, HPLC-MS/MS) results in increased sensitivity, selectivity and ease of detection of multiple toxins in a single step, becoming the dominant instrumentation for mycotoxin analysis in recent years (Straumfors et al. 2015b; Castillo et al. 2016; Niculita-Hirzel et al. 2016; Skóra et al. 2016; Heutte et al. 2017; Schlosser et al. 2020; Szulc et al. 2021, 2022; Ndaw et al. 2021a; b).

The molecular Polymerase Chain Reaction (PCR) technique is applied to identify mycotoxin-producing molds, as surrogates for mycotoxin measurements, but this indirect approach is increasingly less used. This is understandable as the detection of toxigenic species may not, in general, predict mycotoxin presence, as well as the presence of non-mycotoxin-producing fungi, may lead to an overestimation of the predicted mycotoxin concentration. On the other hand, as the mycotoxins may also be present long after the death of the producer, an underestimation of the mycotoxin concentration is also possible (Straumfors 2008).

Various Enzyme-linked immunosorbent assays (ELISA) are are commercially available to detect mycotoxins in human biological fluids (urine and blood). Many authors highlight the importance of combining environmental sampling with biomonitoring to assess also food contribution to the workers’ total exposure.

Concerning urine samples, is emphasized that it is preferable to collect 24-hour urine or first-morning void than to spot samples because they are more concentrated. In the case of DON, there is clear evidence that urinary excretion varies at different times of the day and spot samples cannot describe these differences (Viegas et al. 2019c).

However, biomonitoring alone does not allow to conclude whether the exposure results solely from food intake or whether the working environment is also a contributing factor. Furthermore, this tool needs a control group which enabled to take into account the exposure by food intake and a better understanding of the role of working environments in the total burden of mycotoxin exposure. For these reasons, many authors suggest that using multi-approach sampling (active and passive) and laboratory methods (culture-based, PCR, HPLC, ELISA, cytotoxicity tests, etc.) improved data findings enabling a more detailed and accurate risk assessment (Viegas et al. 2022a).

### Reported health outcomes

Few studies looked for health outcomes resulting from exposure to airborne mycotoxins and most of them are mainly focused on the cytotoxic effects of samples towards cell lines, using the MTT assay (Despot Jakšić et al. 2016; Skóra et al. 2016; Viegas et al. 2017, 2022a, 2022b; Jakšić et al. 2018; Salambanga et al. 2022). Human A549 and HepG2 cell lines are among the most used for lung and liver, respectively (Despot et al. 2016; Jakšić et al. 2018; Viegas et al. 2019c; Szulc et al. 2021; Salambanga et al. 2022). Also Swine kidney (SK) monolayer cells were used as target cells as they are considered the most sensitive in detecting compounds with a known weak cytotoxic activity, like DON (Viegas et al. 2017).

Some authors observed an association between 3-nitropropionic acid and neurologic illnesses in animals and humans (Szulc et al. 2022).

However, only two report actual health outcomes. Lai et al. (2014) observed an elevated risk of hepatocellular carcinoma (HCC) for sugar and papermaking workers with airway exposure to Aspergillus flavus-contaminated dust respect to controls. In similar working environments, low-dose exposure to AFB1 was associated with lung cancer while heavy and prolonged exposure to airway AFB1 may be complicated by the development of lung cancer and HCC (Mo et al. 2014).

### Working areas

#### Animal husbandry

Seven studies have looked for exposure to airborne mycotoxins in breeding farms and animal production plants.

Feeding is the working task that implicates the most exposure and feed (i.e. corn silage, oilseed cakes, hay) has a relevant role as a source of accumulation and release of these contaminants in cattle farms (Lanier et al. 2010, 2012). However, seems that not all mycotoxins (i.e. DON) are aerosolized during their handling, even if present on the feed (Lanier et al. 2012).

Feed handling has turned out to be the most critical task even in pig production plants. In these working environments, high contaminations found in litter (DON: <LOQ–76.4 ng/g; STC: 1.14–2.69 ng/g) and feed samples have made it possible to estimate that feeding, floor sweeping and removal/change of litter will be responsible for the workers’ dust and mycotoxins exposure. In feed samples the common scenario was the co-occurrence of different mycotoxins (9–17 mycotoxins) with higher values for DON (range: 137–388 ng/g) and FB1 (range: 6–366 ng/g). Other metabolites, such as ZEN, 3-AcDON, 15-AcDON, DON-3-G, Fumonisins (FB1, FB2 and FB3), and type A trichothecenes (T-2 and HT-2) were also detected in almost all the feed samples. Therefore, the authors concluded that feed plays a major role in environmental contamination (Viegas et al. 2019c).

#### Agriculture

In agriculture the workers’ exposure can be linked both to the type of plant grown but also to the type of harvesting and processing. Operators involved in the cultivation of cereals, corn and spices are potentially exposed to airborne mycotoxins, especially during the various phases of harvesting, loading and unloading of the vehicles involved in transport, transfer of the crop to drying systems, transfer of the harvest from the storage containers to the processing and treatment plants (sieve, crusher, etc.). In these working environments, certainly grain dust represents the main source of mycotoxin exposure.

Niculita-Hirzel et al. (2016) has reported that the incidence of the mycotoxins differed between activities: wheat harvesting generated on average 28, 20 and 1 ng·m^3^ while grain unloading generated 53, 46 and 4 ng·m^3^ of DON, NIV and ZEN respectively. However, the use of collective protection measures (e.g. working in ventilated cabs) turned out to be very efficient in reducing exposure levels by 10- to 20-fold depending on the activities and the mycotoxin considered. Furthermore, personal samples identified the harvester cleaning as the most critical task exposing grain workers to DON, NIV and ZEN at concentrations as high as 65, 59 and 3 ng·m^3^, followed by the reception of wheat grain to the terminal (16, 7, 1 ng·m^3^). Workers engaged in these activities are often reluctant to wear personal protective equipment above all during the hottest period of the year. The data highlighted that mycotoxins are frequent contaminants of aerosols released during wheat processing confirming their ubiquitous presence in wheat dust and the potential risk of exposure of grain workers, especially to Fusarium mycotoxins.

Similar observations have been reported in Norwegian grain industries where cleaning and the controlling process associated with grain elevators have been identified as strong determinants for increased grain dust exposure (Straumfors et al. 2015a, b).

Cleaning procedure has turned out to be the most critical task also in two different papers conducted in grain elevators. In the first pilot study this activity was associated with high levels of dust (range: 29.7–105 mg m^3^) while mycotoxin exposure levels were highly variable with concentrations between 28.3 and 108 ng m^3^ for DON, 80.0 and 120 pg m^3^ for AFB1, 97.0 and 873 pg m^3^ for FB1, 38.0 and 194 ng m^3^ for OTA and 32.1 and 285 ng m^3^ for ZEN (Ndaw et al. 2021a). The cleaning procedure was the riskiest task also in the second study where workers were highly exposed to airborne organic dust (median 4.92 mg m^3^) and mycotoxins (mainly DON, ZEN and FB1) during these activities. The workers were involved in the cleaning of the empty grain dryers for maize and barley and their main operations consisted of removing any grain or debris that had accumulated and attached to the sides and floor of the dryers. The above results have been confirmed by the urinary DON concentrations that were significantly higher in post-shift than in pre-shift samples (22.1 and 9.9 μg/L respectively). The authors highlighted the usefulness of multi-mycotoxin methods in assessing external and internal exposures which shed light on the extent and pathways of exposure occurring in occupational settings (Ndaw et al. 2021b).

The grinding phase is considered another delicate activity for a possible aerodispersion of dust and fungal metabolites. As reported by Traverso et al. (2010), who performed air samplings during a bulk grinding of peanuts from Vietnam, the wet grinding method proved effective in reducing mycotoxin concentration in the air as they measured negligible levels of AFB1 after grinding (0,11pg/m^3^) than foodstuff concentration before grinding.

A potential risk of exposure to organic dust and fungal metabolites has also been demonstrated in sectors characterized by the handling of flours for human use (bakeries and pizza restaurants) that, as well known, are perfect nutrients for microorganisms growth. In these settings raw materials (e.g. flour) are indoor contamination sources not negligible. The authors emphasized the need for further studies to improve understanding of this setting and develop surveillance and intervention programs aimed at the improvement and protection of the respiratory health of bakery workers (Viegas et al. 2019b, 2020b).

#### Healthcare settings

No or low mycotoxin concentrations (STC 0,31, 0.32,1,45 µg m^3^) were measured in a Hospital and in a Cancer Treatment Center respectively confirming a negligible risk of exposure to these metabolites (Heutte et al. 2017; Viegas et al. 2020a). However, the ability of some fungal species identified to release mycotoxins in vitro (e.g. Aspergillus fumigatus and Aspergillus versicolor) does not entirely rule out a potential health hazard for healthcare personnel. Furthermore, the Heating, Ventilation and Air Conditioning (HVAC) system can be a source of accumulation and release of mycotoxins. In fact, several metabolites were detected in HVAC filter extracts with FB2 the most prevalent compound and with the highest values (0.6–21.4 ng g).

Consequently, factors such as overcrowding, inadequate design, ventilation and the increased use of HVAC without temperature and relative humidity control may boost the growth and dissemination of toxigenic fungi. These results pointed out the need to improve HVAC systems to guarantee indoor environmental quality for patients and workers and support the importance of considering exposure to complex mixtures in indoor environments which is a commonly occurring event.

On the one hand, modern ventilation systems reduce the microbiological contamination of the air, on the other hand, they may get contaminated if not well maintained and become a secondary source of microbial contamination. Thus, it is imperative to implement microbiological monitoring and control measures in these settings, with many countries adopting legislation regarding Indoor Air Quality (IAQ) (Viegas et al. 2019a).

#### Waste and sewage treatment plants

Waste treatment systems provide conditions of moisture and decomposition of organic matter that favour the growth of fungi and are thus considered critical concerning workers’ exposure to fungal metabolites occurring mainly during the handling, lifting and dumping of waste. However, relatively few studies concerning exposure to mycotoxins in this sector have been published although since 2017 there has been increased attention. To date, no or low concentrations were measured suggesting a not significant threat to health.

Molds and mycotoxin contamination in filters from the air conditioning system of forklift cabinets were used as indicators to assess the occupational exposure of the drivers working in a waste sorting facility. Aspergillus species were found most frequently but no mycotoxins were detected in aqueous filter extracts although most extracts were highly or medium cytotoxic. The authors suggest that observing air conditioner filter replacement frequency may be a critical aspect to avoid worker exposure but further research is still needed to check if the environmental conditions, as present in the filters, could allow the production of mycotoxins and their dissemination in the cabinet during the use of the vehicles (Viegas et al. 2017).

Low levels of mycotoxins were measured in another study conducted in mechanical-biological treatment (MBT) and materials recovery (MRF) facilities. In stationary air samples (CIP-10 fixed on a tripod), AFB1 was 0.06 ng m^3^ at the mechanical separation area of MBT plant, while a range of 0.01–0.92 ng m^3^ of STC was quantified at the same area in both facilities. Regarding personal sampling (CIP-10 worn by the workers), AFB1 was detected during compressed-air cleaning task (0.98 ng m^3^) at the mechanical separation area of MBT plant and during manual sorting of newpapers in MRF facility (0.1 ng m^3^). In MRF plant STC was detected both during cleaning (max value: 0.92 ng m^3^) and manual sorting (range: 0.06–0.1 ng m^3^) tasks. For both compounds, the highest mycotoxin value (personal sampling) was associated with the highest level of inhalable dust, which occurred during cleaning tasks (AFB1/inhalable dust: 0.98 ng m^3^/35.8 mg m^3^ at the MBT plant; STC/inhalable dust: 0.92 ng m^3^/28.0 mg m^3^ at the MRF plant) (Schlosser et al. 2020).

In the study of Karamkhani et al. (2020) surfaces, personal, air dust and blood samples were collected from the plastic and bread waste-sorting sections in three recycling municipal dry waste sites. Authors reported that operators engaged in the bread waste sorting were at the greatest risk of exposure (4.5–15.1 ng m^3^ of AFB1 in a working week) and, in general, workers handling municipal waste may be exposed to hazardous levels of AFB1. These results were confirmed by biomonitoring tool: AFB1-Alb was significantly higher in the exposed workers, especially in the bread sorting section, as compared to controls.

#### Other occupational environments

An interesting paper reported that documentary material handled in libraries and archives (e.g. antique books) can be contaminated by fungal strains mycotoxin-producers with allergenic properties (Castillo et al. 2016). This detection may represent a potential risk for researchers and other personnel indicating the need to help strengthen security measures and monitor this occupational sector until more data are available.

## Conclusions

This review provides an overview of the occurrence of airborne mycotoxins in working environments from 2010 to date.

A consistent number of papers measured and confirmed existing of mycotoxins in environmental samples, however, the nature of compounds and the magnitude of exposure vary depending on the working environment, the products handled or the tasks performed by workers.

Our review shows that the most common scenario is the exposure to several mycotoxins so many authors consider that it is important to provide multi-approach sampling and analytical protocols to achieve an accurate and more realistic risk characterization.

The most exposed to airborne mycotoxins are workers engaged in animal husbandry and, in particular, those designated to feeding tasks. During these processes, the wearing of personal protective equipment, especially respiratory protective devices (FFP2), is largely encouraged. An additional preventive action can be the choice of raw materials used during feed formulation. Considering this aspect, the geographic origin of the raw material can have a great influence on the mycotoxin contamination of feed at different stages of production.

In agriculture harvester cleaning is the procedure with the highest level of exposure. Unfortunately, the workers assigned to this task are often reluctant to wear personal protective equipment during the hottest and the most stressful period of the year.

Data concerning exposure in waste/sewage treatments plants are still unclear. The cleaning procedure seems to be the riskiest task but additional data are required to confirm this assumption.

The detection of mycotoxin-producing molds in healthcare environments and the role of the HVAC system as a source of microbial contamination reinforce the relevance of studying more these settings paying special attention to the maintenance and regular replacement of filters.

With regard to health outcomes, to date epidemiological studies are insufficient to provide a clear picture of the health risks related to mycotoxin exposure by inhalation. This is particularly challenging since one mycotoxin can elicit more than one type of effects and these can occur at different exposure level.

Further field investigations are needed to support our considerations and to identify other sectors and/or working tasks at risk of exposure to mycotoxins via inhalation. This information is crucial for hygienists and occupational technicians in order to monitor and implement prevention and control strategies. In this contest researchers should work together to select/develop standardised sampling and analysis methodologies and participate in large-scale studies to obtain relevant data.

## Supplementary Material

wxad070_suppl_Supplementary_Figure_S1

## Data Availability

All data that support the findings of this study are available after the reasonable request to the corresponding author.

## References

[CIT0001] Al-Jaal BA , Jaganjac M, Barcaru A, Horvatovich P, Latiff A. Aflatoxin, fumonisin, ochratoxin, zearalenone and deoxynivalenol biomarkers in human biological fluids: a systematic literature review, 2001–2018. Food Chem Toxicol. 2019:129:211–228. 10.1016/j.fct.2019.04.047 31034935

[CIT0002] Awuchi CG , Ondari EN, Nwozo S, Odongo GA, Eseoghene IJ, Twinomuhwezi H, Ogbonna CU, Upadhyay AK, Adeleye AO, Okpala COR. Mycotoxins’ toxicological mechanisms involving humans, livestock and their associated health concerns: A review. Toxins (Basel). 2022:14(3):167. 10.3390/toxins14030167 35324664 PMC8949390

[CIT0003] Boonen J , Malysheva SV, Taevernier L, Diana D, Mavungu J, De Saeger S, De Spiegeleer B. Human skin penetration of selected model mycotoxins. Toxicology. 2012:301(1–3):21–32. 10.1016/j.tox.2012.06.012 22749975

[CIT0004] Brasel TL , Douglas DR, Wilson SC, Straus DC. Detection of airborne Stachybotrys chartarum macrocyclic trichothecene mycotoxins on particulates smaller than conidia. Appl Environ Microbiol. 2005:71(1):114–122. 10.1128/AEM.71.1.114-122.2005 15640178 PMC544211

[CIT0005] Castillo NI , Ibáñez M, Beltrán E, Rivera-Monroy J, Ochoa JC, Páez-Castillo M, Posada-Buitrago ML, Sulyok M, Hernández F. Identification of mycotoxins by UHPLC–QTOF MS in airborne fungi and fungi isolated from industrial paper and antique documents from the Archive of Bogotá. Environ Res. 2016:144(Pt A):130–138. 10.1016/j.envres.2015.10.031 26599591

[CIT0006] Commission Regulation (EC) No. 1881/2006. Setting maximum levels for certain contaminants in foodstuffs. https://eurlex.europa.eu/legal-content/IT/TXT/PDF/?uri=CELEX:32006R1881&from=ES

[CIT0007] Daou R , Joubrane K, Maroun RG, Khabbaz LR, Ismail A, Khoury AE. Mycotoxins: factors influencing production and control strategies. AIMS Agric Food. 2021:6:416–447. 10.3934/agrfood.2021025

[CIT0008] Despot Jakšić D , Kocsubé S, Bencsik O, Kecskeméti A, Szekeres A, Vágvölgyi C, Varga J, Klarić MS. Species diversity and cytotoxic potency of airborne sterigmatocystin-producing aspergilli from the section versicolores. Sci Total Environ. 2016:562:296–304. 10.1016/j.scitotenv.2016.03.183 27100010

[CIT0009] Ferri F , Brera C, De Santis B, Fedrizzi G, Bacci T, Bedogni L, Capanni S, Collini G, Crespi E, Debegnach F, et al. Survey on urinary levels of aflatoxins in professionally exposed workers. Toxins. 2017:9(4):117. 10.3390/toxins9040117 28338636 PMC5408191

[CIT0010] Franco LT , Oliveira CAF. Assessment of occupational and dietary exposures of feed handling workers to mycotoxins in rural areas from São Paulo, Brazil. Sci Total Environ. 2022:837:155763. 10.1016/j.scitotenv.2022.155763 35561905

[CIT0011] Gareis M , Gottschalk C. Stachybotrys spp and the guttation phenomenon. Mycotoxin Res. 2014:30(3):151–159. 10.1007/s12550-014-0193-3 24619360

[CIT0012] Guerre P. Worldwide mycotoxins exposure in pig and poultry feed formulations. Toxins. 2016:8(12):350. 10.3390/toxins8120350 27886128 PMC5198545

[CIT0013] Haque MA , Wang Y, Shen Z, Li X, Saleemi MK, He C. Mycotoxin contamination and control strategy in human, domestic animal and poultry: A review. Microb Pathog. 2020:142:104095. 10.1016/j.micpath.2020.104095 32097745

[CIT0014] Heutte N , André V, Dubos Arvis C, Bouchart V, Lemarié F, Legendre P, Votier E, Louis MY, Madelaine S, Séguin V, et al. Assessment of multi-contaminant exposure in a cancer treatment center: a 2-year monitoring of molds, mycotoxins, endotoxins, and glucans in bioaerosols. Environ Monit Assess. 2017:189(1):31. 10.1007/s10661-016-5751-z 28012082

[CIT0015] Huttunen K , Korkalainen M. Microbial secondary metabolites and knowledge on inhalation effects. In: Viegas C, Viegas S, Gomes A, Täubel M, Sabino R, editors. Exposure to microbiological agents in indoor and occupational environments. Cham: Springer; 2017. p. 213–234. 10.1007/978-3-319-61688-9

[CIT0016] Jakšić D , Kocsubé S, Bencsik O, Kecskeméti A, Szekeres A, Jelić D, Kopjar N, Vágvölgyi C, Varga J, Šegvić Klarić M. Fumonisin production and toxic capacity in airborne black Aspergilli. Toxicol In Vitro. 2018:53:160–171. 10.1016/j.tiv.2018.08.006 30149077

[CIT0017] Karamkhani M , Asilian-Mahabadi H, Daraei B, Seidkhani-Nahal A, Noori-Zadeh A. Liver and kidney serum profile abnormalities in workers exposed to aflatoxin B1 in urban solid waste management centers. Environ Monit Assess. 2020:192(7):472. 10.1007/s10661-020-08422-y 32607657

[CIT0018] Kraft S , Buchenauer L, Polte T. Mold, mycotoxins and a dysregulated immune system: A combination of concern? Int J Mol Sci. 2021:22(22):12269. 10.3390/ijms222212269 34830149 PMC8619365

[CIT0019] Krysinska-Traczyk E , Perkowski J, Dutkiewicz J. Levels of fungi and mycotoxins in the samples of grain and grain dust collected from five various cereal crops in eastern Poland. Ann Agric Environ Med. 2007:14(1):159–167. PMID: 17655194. 17655194

[CIT0020] Lai H , Mo X, Yang Y, He K, Xiao J, Liu C, Chen J, Lin Y. Association between aflatoxin B1 occupational airway exposure and risk of hepatocellular carcinoma: a case-control study. Tumour Biol. 2014:35(10):9577–9584. 10.1007/s13277-014-2231-3 24961349 PMC4213372

[CIT0021] Lanier C , André V, Séguin V, Heutte N, El Kaddoumi A, Bouchart V, Picquet R, Garon D. Recurrence of Stachybotrys chartarum during mycological and toxicological study of bioaerosols collected in a dairy cattle shed. Ann Agric Environ Med. 2012:19(1):61–67. PMID: 22462447. 22462447

[CIT0022] Lanier C , Richard E, Heutte N, Picquet R, Bouchart V, Garon D. Airborne molds and mycotoxins associated with handling of corn silage and oilseed cakes in agricultural environment. Atmos Environ. 2010:44(16):1980–1986. 10.1016/j.atmosenv.2010.02.040

[CIT0023] List of Classifications – IARC Monographs on the Identification of Carcinogenic Hazards to Humans. [accessed 2023 May 15]. https://monographs.iarc.who.int/list-of-classifications

[CIT0024] Mo X , Lai H, Yang Y, Xiao J, He K, Liu C, Chen J, Lin Y. How dose airway exposure to aflatoxin B1 affect serum albumin adduct concentrations? Evidence based on epidemiological study and animal experimentation. J Toxicol Sci. 2014:39(4):645–653. 10.2131/jts.39.645 25056789

[CIT0025] Ndaw S , Jargot D, Antoine G, Denis F, Melin S, Robert A. Investigating multi-mycotoxin exposure in occupational settings: A biomonitoring and airborne measurement approach. Toxins. 2021a:13(1):54. 10.3390/toxins13010054 33450876 PMC7828332

[CIT0026] Ndaw S , Remy A, Jargot D, Antoine G, Denis F, Robert A. Mycotoxins exposure of french grain elevator workers: Biomonitoring and airborne measurements. Toxins. 2021b:13(6):382. 10.3390/toxins13060382 34071776 PMC8229223

[CIT0027] Niculita-Hirzel H , Hantier G, Storti F, Plateel G, Roger T. Frequent occupational exposure to fusarium mycotoxins of workers in the swiss grain industry. Toxins. 2016:8(12):370. 10.3390/toxins8120370 27973454 PMC5198564

[CIT0029] Ráduly Z , Szabó L, Madar A, Pócsi I, Csernoch L. Toxicological and medical aspects of aspergillus-derived mycotoxins entering the feed and food chain. Front Microbiol. 2020:10:2908. 10.3389/fmicb.2019.02908 31998250 PMC6962185

[CIT0030] Salambanga FRD , Wingert L, Valois I, Lacombe N, Gouin F, Trépanier J, Debia M, Soszczyńska E, Twarużek M, Kosicki R, et al. Microbial contamination and metabolite exposure assessment during waste and recyclable material collection. Environ Res. 2022:212(Part D):113597. 10.1016/j.envres.2022.113597 35660405

[CIT0031] Schlosser O , Robert S, Noyon N. Airborne mycotoxins in waste recycling and recovery facilities: Occupational exposure and health risk assessment. Waste Manag. 2020:105:395–404. 10.1016/j.wasman.2020.02.031 32126367

[CIT0032] Skóra J , Matusiak K, Wojewódzki P, Nowak A, Sulyok M, Ligocka A, Okrasa M, Hermann J, Gutarowska B. Evaluation of microbiological and chemical contaminants in poultry farms. Int J Environ Res Public Health. 2016:13(2):192. 10.3390/ijerph13020192 26861361 PMC4772212

[CIT0033] Straumfors A , Heldal KK, Wouters IM, Eduard W. Work tasks as determinants of grain dust and microbial exposure in the norwegian grain and compound feed industry. Ann Occup Hyg. 2015a:59(6):724–736. 10.1093/annhyg/mev012 25743566

[CIT0034] Straumfors A , Uhlig S, Eriksen GS, Heldal KK, Eduard W, Krska R, Sulyok M. Mycotoxins and other fungal metabolites in grain dust from Norwegian grain elevators and compound feed mills. World Mycotoxin J. 2015b:8(3):361–373. 10.3920/WMJ2014.1799

[CIT0035] Straumfors Halstensen A. Species-specific fungal DNA in airborne dust as surrogate for occupational mycotoxin exposure? Int J Mol Sci. 2008:9(12):2543–2558. 10.3390/ijms9122543 19330091 PMC2635655

[CIT0036] Szulc J , Okrasa M, Majchrzycka K, Sulyok M, Nowak A, Ruman T, Nizioł J, Szponar B, Gutarowska B. Microbiological and toxicological hazards in sewage treatment plant bioaerosol and dust. Toxins. 2021:13(10):691. 10.3390/toxins13100691 34678984 PMC8540054

[CIT0037] Szulc J , Okrasa M, Majchrzycka K, Sulyok M, Nowak A, Szponar B, Górczyńska A, Ryngajłło M, Gutarowska B. Microbiological and toxicological hazard assessment in a waste sorting plant and proper respiratory protection. J Environ Manage. 2022:303:114257. 10.1016/j.jenvman.2021.114257 34920354

[CIT0038] Traverso A , Bassoli V, Cioè A, Anselmo S, Ferro M. Assessment of aflatoxin exposure of laboratory worker during food contamination analyses Assessment of the procedures adopted by an ARPAL laboratory (Liguria Region Environmental Protection Agency). Med Lav. 2010:101(5):375–380. PMID: 21105592. 21105592

[CIT0039] Viegas C , Almeida B, Monteiro A, Aranha Caetano AL, Carolino E, Quintal Gomes A, Twarużek M, Kosicki R, Marchand G, Viegas S. Bioburden in health care centers: Is the compliance with Portuguese legislation enough to prevent and control infection? Build Environ. 2019a:160:106226. 10.1016/j.buildenv.2019.106226

[CIT0040] Viegas C , Almeida B, Monteiro A, Paciência I, Rufo J, Aguiar L, Lage B, Diogo Gonçalves LM, Caetano LA, Carolino E, et al. Exposure assessment in one central hospital: A multi-approach protocol to achieve an accurate risk characterization. Environ Res. 2020a:181:108947. 10.1016/j.envres.2019.108947 31767353

[CIT0041] Viegas S , Assunção R, Martins C, Nunes C, Osteresch B, Twarużek M, Kosicki R, Grajewski J, Ribeiro E, Viegas C. Occupational exposure to mycotoxins in swine production: Environmental and biological monitoring approaches. Toxins. 2019c:11(2):78. 10.3390/toxins11020078 30717100 PMC6410041

[CIT0042] Viegas C , Faria T, Caetano LA, Carolino E, Quintal-Gomes A, Twarużek M, Kosicki R, Viegas S. Characterization of occupational exposure to fungal burden in Portuguese bakeries. Microorganisms. 2019b:7(8):234. 10.3390/microoganisms7080234 31382481 PMC6723507

[CIT0043] Viegas C , Faria T, de Oliveira AC, Caetano LA, Carolino E, Quintal-Gomes A, Twarużek M, Kosicki R, Soszczyńska E, Viegas S. A new approach to assess occupational exposure to airborne fungal contamination and mycotoxins of forklift drivers in waste sorting facilities. Mycotoxin Res. 2017:33(4):285–295. 10.1007/s12550-017-0288-8 28730564

[CIT0044] Viegas C , Fleming GTA, Kadir A, Almeida B, Caetano LA, Quintal Gomes A, Twarużek M, Kosicki R, Viegas S, Coggins AM. Occupational exposures to organic dust in Irish bakeries and a pizzeria restaurant. Microorganisms. 2020b:8(1):118. 10.3390/microorganisms8010118 31952269 PMC7022993

[CIT0045] Viegas C , Gomes B, Pimenta R, Dias M, Cervantes R, Caetano LA, Carolino E, Twaruzek M, Soszczynska E, Kosicki R, et al. Microbial contamination in firefighter headquarters’: A neglected occupational exposure scenario. Build Environ. 2022a:213:108862. 10.1016/j.buildenv.2022.108862 35483278

[CIT0046] Viegas C , Pena P, Dias M, Gomes B, Cervantes R, Carolino E, Twarużek M, Soszczyńska E, Kosicki R, Caetano LA, et al. Microbial contamination in waste collection: Unveiling this Portuguese occupational exposure scenario. J Environ Manage. 2022b:314:115086. 10.1016/j.jenvman.2022.115086 35483278

[CIT0047] Viegas S , Veiga L, Figueredo P, Almeida A, Carolino E, Sabino R, Veríssimo C, Viegas C. Occupational exposure to aflatoxin B1 in swine production and possible contamination sources. J Toxicol Environ Health A. 2013:76(15):944–951. 10.1080/15287394.2013.826569 24156697

[CIT0048] Viegas S , Veiga L, Malta-Vacas J, Sabino R, Figueredo P, Almeida A, Viegas C, Carolino E. Occupational exposure to aflatoxin (AFB_1_) in poultry production. J Toxicol Environ Health A. 2012:75(22–23):1330–1340. 10.1080/15287394.2012.721164 23095151

[CIT0049] Wang Y , Zhao C, Zhang D, Zhao M, Zheng D, Peng M, Cheng W, Guo P, Cui Z. Simultaneous degradation of aflatoxin B1 and zearalenone by a microbial consortium. Toxicon. 2018:146:69–76. 10.1016/j.toxicon.2018.04.007 29621525

[CIT0050] World Health Organization (WHO). [accessed 2023 March 9]. https://www.who.int/news-room/fact-sheets/detail/mycotoxins

