# Supplementary material for: Exposure to airborne mycotoxins: the riskiest working environments and tasks
Source: Ann Work Expo Health. 2023 Nov 28;68(1):19–35. doi: 10.1093/annweh/wxad070 (PMC10773202; doi:10.1093/annweh/wxad070)
Supplement: wxad070_suppl_Supplementary_Figure_S1 [file wxad070_suppl_supplementary_figure_s1.pdf]

Exposure to airborne mycotoxins: the riskiest working environments and tasks

Anna Maria Marcelloni[1], Daniela Pigini[1], Alessandra Chiominto[1], Angela Gioffrè[2] and Emilia Paba[1,*]

**PubMed**	(("mycotoxins"[MeSH Terms] OR "mycotoxins"[All Fields] OR "mycotoxin"[All Fields] OR (("fungals"[All Fields] OR "microbiology"[MeSH Terms] OR "microbiology"[All Fields] OR "fungal"[All Fields] OR "fungi"[MeSH Terms] OR "fungi"[All Fields]) AND ("metabolite"[All Fields] OR "metabolite s"[All Fields] OR "metabolites"[All Fields]))) AND ("administration, inhalation"[MeSH Terms] OR ("administration"[All Fields] AND "inhalation"[All Fields]) OR "inhalation administration"[All Fields] OR "inhalant"[All Fields] OR "inhalability"[All Fields] OR "inhalable"[All Fields] OR "inhalants"[All Fields] OR "inhalated"[All Fields] OR "inhalation"[MeSH Terms] OR "inhalation"[All Fields] OR "inhal"[All Fields] OR "inhalations"[All Fields] OR "inhale"[All Fields] OR "inhaled"[All Fields] OR "inhaling"[All Fields] OR "inhalational"[All Fields] OR "inhalative"[All Fields] OR "inhalatively"[All Fields] OR "inhalent"[All Fields] OR "inhaler s"[All Fields] OR "inhales"[All Fields] OR "nebulizers and vaporizers"[MeSH Terms] OR ("nebulizers"[All Fields] AND "vaporizers"[All Fields]) OR "nebulizers and vaporizers"[All Fields] OR "inhalator"[All Fields] OR "inhalators"[All Fields] OR "inhaler"[All Fields] OR "inhalers"[All Fields] OR ("inhalation exposure"[MeSH Terms] OR ("inhalation"[All Fields] AND "exposure"[All Fields]) OR "inhalation exposure"[All Fields]) OR ("air"[MeSH Terms] OR "air"[All Fields])) AND ("workplace"[MeSH Terms] OR "workplace"[All Fields] OR "workplaces"[All Fields] OR "workplace s"[All Fields] OR ("working conditions"[MeSH Terms] OR ("working"[All Fields] AND "conditions"[All Fields]) OR "working conditions"[All Fields]) OR ("occupational groups"[MeSH Terms] OR ("occupational"[All Fields] AND "groups"[All Fields]) OR "occupational groups"[All Fields]) OR ("occupational exposure"[MeSH Terms] OR ("occupational"[All Fields] AND "exposure"[All Fields]) OR "occupational exposure"[All Fields]) OR (("occupant"[All Fields] OR "occupant s"[All Fields] OR "occupants"[All Fields] OR "occupational"[All Fields] OR "occupations"[MeSH Terms] OR "occupations"[All Fields] OR "occupation"[All Fields]) AND ("environ"[All Fields] OR "environment"[MeSH Terms] OR "environment"[All Fields] OR "environments"[All Fields] OR "environment s"[All Fields] OR "environs"[All Fields])))) AND (2010/1/1:2023/5/15[pdat]) AND english[Language])
**Web of Science**	((AB=(mycotoxins OR fungal metabolites)) AND AB=(inhalation OR inhalation exposure OR air)) AND AB=(workplace OR working conditions OR occupational groups OR occupational exposure OR occupational environment) and Review Article (Exclude – Document Types) and Timespan: 2010-01-01 to 2023-05-15 (Publication Date)
**Scopus**	TITLE-ABS-KEY (mycotoxins) AND ALL (inhalation OR airborne OR air) AND ABS (workplace OR workers OR occupational OR environment) AND (LIMIT-TO (DOCTYPE , "ar") OR EXCLUDE (DOCTYPE , "ch") OR EXCLUDE (DOCTYPE , "re") OR EXCLUDE (DOCTYPE , "bk") OR EXCLUDE (DOCTYPE , "cp")) AND (LIMIT-TO (PUBYEAR , 2023) OR LIMIT-TO (PUBYEAR , 2022) OR LIMIT-TO (PUBYEAR , 2021) OR LIMIT-TO (PUBYEAR , 2020) OR LIMIT-TO (PUBYEAR , 2019) OR LIMIT-TO (PUBYEAR , 2018) OR LIMIT-TO (PUBYEAR , 2017) OR LIMIT-TO (PUBYEAR , 2016) OR LIMIT-TO (PUBYEAR , 2015) OR LIMIT-TO (PUBYEAR , 2014) OR LIMIT-TO (PUBYEAR , 2013) OR LIMIT-TO (PUBYEAR , 2012) OR LIMIT-TO (PUBYEAR , 2011) OR LIMIT-TO (PUBYEAR , 2010)) AND (LIMIT-TO (LANGUAGE , "English")) AND (LIMIT-TO (PUBSTAGE , "final"))

Figure S1: Search strings used in the literature search
